# *DPYD*, *TYMS*, *TYMP*, *TK1*, and *TK2* Genetic Expressions as Response Markers in Locally Advanced Rectal Cancer Patients Treated with Fluoropyrimidine-Based Chemoradiotherapy

**DOI:** 10.1155/2013/931028

**Published:** 2013-12-23

**Authors:** Ming-Yii Huang, Chan-Han Wu, Chun-Ming Huang, Fu-Yen Chung, Ching-Wen Huang, Hsiang-Lin Tsai, Chin-Fan Chen, Shiu-Ru Lin, Jaw-Yuan Wang

**Affiliations:** ^1^Department of Radiation Oncology, Kaohsiung Medical University Hospital, Kaohsiung Medical University, Kaohsiung 80708, Taiwan; ^2^Department of Radiation Oncology, Faculty of Medicine, College of Medicine, Kaohsiung Medical University, Kaohsiung 80708, Taiwan; ^3^Cancer Center, Kaohsiung Medical University Hospital, Kaohsiung Medical University, Kaohsiung 80708, Taiwan; ^4^Department of Medical Research, Fooyin University Hospital, Pingtung County 928, Taiwan; ^5^Graduate Institute of Medicine, College of Medicine, Kaohsiung Medical University, Kaohsiung 80708, Taiwan; ^6^Division of Gastrointestinal and General Surgery, Department of Surgery, Kaohsiung Medical University Hospital, Kaohsiung 80708, Taiwan; ^7^Department of Surgery, Kaohsiung Municipal Hsiao-Kang Hospital, Kaohsiung 812, Taiwan; ^8^Division of General Surgery Medicine, Department of Surgery, Kaohsiung Medical University Hospital, Kaohsiung 80708, Taiwan; ^9^Graduate Institute of Clinical Medicine, College of Medicine, Kaohsiung Medical University, Kaohsiung 80708, Taiwan; ^10^Department of Surgery, Faculty of Medicine, College of Medicine, Kaohsiung Medical University, Kaohsiung 80708, Taiwan; ^11^Department of Genomic Medicine, College of Medicine, Kaohsiung Medical University, Kaohsiung 80708, Taiwan

## Abstract

This study is to investigate multiple chemotherapeutic agent- and radiation-related genetic biomarkers in locally advanced rectal cancer (LARC) patients following fluoropyrimidine-based concurrent chemoradiotherapy (CCRT) for response prediction. We initially selected 6 fluoropyrimidine metabolism-related genes (*DPYD, ORPT, TYMS, TYMP, TK1*, and *TK2*) and 3 radiotherapy response-related genes (*GLUT1*, *HIF-1* **α**, and *HIF-2* **α**) as targets for gene expression identification in 60 LARC cancer specimens. Subsequently, a high-sensitivity weighted enzymatic chip array was designed and constructed to predict responses following CCRT. After CCRT, 39 of 60 (65%) LARC patients were classified as responders (pathological tumor regression grade 2 ~ 4). Using a panel of multiple genetic biomarkers (chip), including *DPYD*, *TYMS*, *TYMP*, *TK1*, and *TK2*, at a cutoff value for 3 positive genes, a sensitivity of 89.7% and a specificity of 81% were obtained (AUC: 0.915; 95% CI: 0.840–0.991). Negative chip results were significantly correlated to poor CCRT responses (TRG 0-1) (*P* = 0.014, hazard ratio: 22.704, 95% CI: 3.055–235.448 in multivariate analysis). Disease-free survival analysis showed significantly better survival rate in patients with positive chip results (*P* = 0.0001). We suggest that a chip including *DPYD*, *TYMS*, *TYMP*, *TK1*, and *TK2* genes is a potential tool to predict response in LARC following fluoropyrimidine-based CCRT.

## 1. Introduction

Colorectal cancer (CRC) is the third most common malignancy, and morbidity and mortality due to CRC are increasing worldwide [1]. Despite substantial progress in both diagnosis and therapy in recent decades, the prognosis for CRC remains poor. Approximately 35–40% of patients with locally advanced rectal cancer (LARC) will eventually develop distant metastases and die from this disease [2]. One of the leading causes of rectal cancer-related death is therapy resistance [3]. In locally advanced stages of rectal cancer, clinical outcomes can be improved by preoperative neoadjuvant radiation or concurrent chemoradiotherapy (CCRT). Preoperative CCRT, introduced in the past decade, can achieve better sphincter preservation rates and lower local recurrence rates and can downstage the disease. It has therefore become a consensus treatment modality for LARC [4–8]. Although complete pathological response rates of 10–25% can be achieved, more than one-third of patients either do not respond or show only modest response to treatment [6]. The rate of local recurrence or distant metastasis remains as high as 15–20% for LARC treated with preoperative CCRT [8, 9]. The disease-free survival (DFS) of rectal cancer patients receiving preoperative CCRT with tumor response is better than that of patients with progressive or stable disease [7, 10].

The response of individual tumors to adjuvant therapies is not uniform. This poses a considerable clinical dilemma because patients with *a priori* resistant tumors could be spared exposure to radiation or DNA-damaging drugs, treatments that are associated with substantial adverse effects, and surgery could be scheduled without delay. Alternatively, different adjuvant treatment modalities, including additional chemotherapeutics, could be pursued. Therefore, it would be of significant clinical relevance to identify predictive biomarkers of response in LARC following CCRT.

Accordingly, several studies have investigated the correlation of various gene expression levels and tumor responses to different chemotherapeutic drugs, radiotherapy, and CCRT; however, the predictive value of at least some of these markers remains controversial [11–17]. For instance, thymidylate synthetase (*TYMS*) and other fluorouracil-associated enzymes (such as thymidine phosphorylase (*TYMP*) and dihydropyrimidine dehydrogenase (*DPYD*)) have been analyzed with respect to the local recurrence and development of metastasis of CRC after postoperative 5-fluorouracil (5-FU) chemotherapy [18]. Overexpression of *TYMS* is associated with resistance to 5-FU chemotherapy and can lead to poorer CRC survival rates, both DFS and overall survival (OS) [19]. Traditionally, the methodology used to identify predictive factors for response to fluoropyrimidine-based treatments has been to analyze the expression of enzymes implicated in its metabolism, either directly by immunohistochemistry (IHC) or by an enzyme-linked immune-sorbent assay (ELISA) or indirectly by individual mRNA expression [14, 20, 21]. More recently, the development of high-throughput methods of multiple genetic expression analysis has enabled a broader approach, analyzing multiple genes profiles simultaneously and providing genomic response signatures.

Conventional regimens for treating cancer patients with chemotherapy and radiotherapy do not account for inter-patient variability in the expression of particular target genes. Such variability results in unpredictable tumor responses and host toxicity. Hence, our study investigated the role of the genetic expression levels of 6 fluoropyrimidine-based chemotherapy-related genes (*DPYD*, *TYMS*, *TYMP*, thymidine kinase 1, soluble (*TK1*), thymidine kinase 2, mitochondrial (*TK2*), and orotate phosphoribosyl transferase (*ORPT*)) and 3 genes related to radiotherapy (RT) response (glucose transporter member 1 (*GLUT1*), hypoxia-inducible factor 1 (*HIF1*), and hypoxia-inducible factor 2 (*HIF2*)) in the literature, genomic databases, and the Medline database [22–28].

Previously, our laboratory has successfully established a weighted enzymatic chip array (WEnCA) platform that could identify candidate genes as predictive biomarkers for potential clinical implications [29]. In the current study, we collected preoperative CCRT tumor tissues and paired normal tissues from 60 LARC patients. The correlations between the gene expression levels of the 9 candidate genes and the clinicopathological features of LARC patients, in addition to the relationship between gene expression levels and the CCRT tumor response, were analyzed to elucidate the role of a panel of multiple genetic biomarkers as a predictor of tumor response in LARC patients following preoperative CCRT.

This is the first investigation regarding predicting the clinical outcome of CCRT using a panel of multiple genetic biomarkers for LARC patients. The results would have potential clinical implications for predicting which patients would be likely to respond to preoperative CCRT and those who would be unlikely to respond, for whom therapeutic strategies would probably be altered.

## 2. Materials and Methods

### 2.1. Patients and Samples Collection

Between November 2006 and June 2011, 60 patients with LARC (T3/T4 disease or any clinical positive N-stage) located within 10 cm of the anal verge and receiving fluoropyrimidine-based preoperative CCRT were enrolled in this study. The study was approved by the ethics committee of our hospital. Baseline assessment before initiation of CCRT included a complete medical history and physical examination, colonoscopy, tumor biopsy, pelvic and abdominal computed tomography (CT), endorectal ultrasonography (if clinically feasible), and/or pelvic magnetic resonance imaging. Complete laboratory tests included a complete blood cell count, liver function tests, electrolytes, creatinine, albumin, and carcinoembryonic antigen (CEA). All patients had Eastern Cooperative Oncology Group (ECOG) performance status <2, were between 18 and 85 years of age, and had adequate hematological, liver, and renal function. Each tissue sample was snap-frozen in liquid nitrogen immediately after surgery or biopsy and stored at −80°C. Samples were further used in experiments for membrane array analysis. Clinical stage and pathological features of primary tumors were defined according to the criteria of the American Joint Commission on Cancer/International Union Against Cancer (AJCC/UICC) [30].

### 2.2. Treatments

Patients were treated with fluoropyrimidine-based chemotherapy. Of the 60 patients, 24 were treated with 5-fluorouracil (5-FU) (350 mg/m^2^ IV bolus) and leucovorin (20 mg/m^2^ IV bolus) with the fractions of the radiotherapy being administered on days 1 through 5 and days 21 through 25. Thirty-six patients were treated with capecitabine (850 mg/m^2^, twice daily, 5 days a week, during the days when radiotherapy was administered). The first daily dose of capecitabine was given 2 hours before radiotherapy; the second dose was administered 8–10 hours later. Radiotherapy (RT) was planned via computerized dosimetry, and a dose of 1.8 Gy per fraction was prescribed to cover the planned target volume. Pelvic RT consisted of 45 Gy in 25 fractions over a period of 5 weeks. The clinical target volume contained the primary tumor, the mesorectum, the presacral space, and the lymph nodes, which included the perirectal, presacral, internal iliac, and/or external iliac nodes. Patients were evaluated weekly during the course of CCRT to assess acute toxicity and their own compliance with the study. Blood tests were performed each time and consisted of complete blood cell and differential counts. The toxicity was monitored by use of the National Cancer Institute Common Toxicity Criteria, version 3.0 (http://ctep.cancer.gov/reporting/ctc.html; accessed in December 2012). Chemotherapy was withheld if any chemotherapy-related grade 3 or 4 toxicity was noted, in which case appropriate dose adjustment was undertaken. Chemotherapy was restarted at an 80% dose if toxicity levels resolved and was terminated if grade 3 or 4 toxicity was noted again after adjustment of the dosage. If grade 3 or 4 toxicity was clearly related to RT (e.g., with radiation dermatitis), local therapy was administered and chemotherapy was not terminated. After completion of the CCRT, all patients underwent surgery with a total mesorectal excision (TME), and extended visceral resection was performed in the clinical T4 patients. All operations were carried out by a single colorectal surgery specialist (J.-Y. Wang), who had performed more than 300 TMEs in the past 5 years. Anal sphincter-sparing surgery was performed whenever possible, with primary anastomosis and/or temporarily diverting colostomies.

### 2.3. Tumor Response

The characteristics of each LARC patient, any adverse events, and their responses after the CCRT were recorded. Assessment of pathological tumor response to preoperative CCRT was based on a standardized tumor regression grading (TRG) as described by Dworak et al. [31]. Two pathologists were involved in this study. They were blinded to the results of the array and scored each specimen independently. Any specimen where a difference in scores existed was then scored by consensus using a double-headed microscope. TRG was determined by the amount of viable tumor versus fibrosis, ranging from TRG 4 (no viable tumor cells detected) to TRG 0 (fibrosis completely absent). TRG 3 was defined as a regression of more than 50% with fibrosis outgrowing the tumor mass; TRG 2 was defined as a regression of less than 50%, and TRG 1 was basically defined as a morphologically unaltered tumor mass. In this study, pathological tumor response was defined as ranging between TRG 2 and TRG 4. The determination for downstaging was based on the comparison between the clinical TNM stage before the initiation of CCRT and the postoperative histopathological TNM stage.

### 2.4. Total RNA Extraction and First-Strand cDNA Synthesis

Total RNA was isolated from each LARC patient's tissue with the GeneCling Enzymatic Gene Chip Detection Kit (MedicoGene Biotechnology Co., Ltd., LA, USA). RNA purified was quantified by measuring absorption at OD 260 nm using an ND-1000 spectrophotometer (NanoDrop Technologies, Wilmington, DE, USA) and quantitated by Bioanalyzer 2100 (Agilent Technologies, Santa Clara, CA, USA). First-strand cDNA was synthesized from total RNA, using the GeneCling Enzymatic Gene Chip Detection Kit. Reverse transcription was carried out in a reaction mixture consisting of 3 *μ*g/mL oligo (dT) 18-mer primer, 1 *μ*g/mL random 6-mer primer, 100 mmol/L deoxyribonucleotide triphosphate, 200 units of MMLV reverse transcriptase, and 25 units of ribonuclease inhibitor. The reaction mixtures with RNA were incubated at 42°C for a minimum of 2 hours, heated to 95°C for 5 minutes, and then stored at −80°C until analysis.

### 2.5. Preparation of Biotin-Labeled cDNA Targets and Hybridization

**Figure 1:**
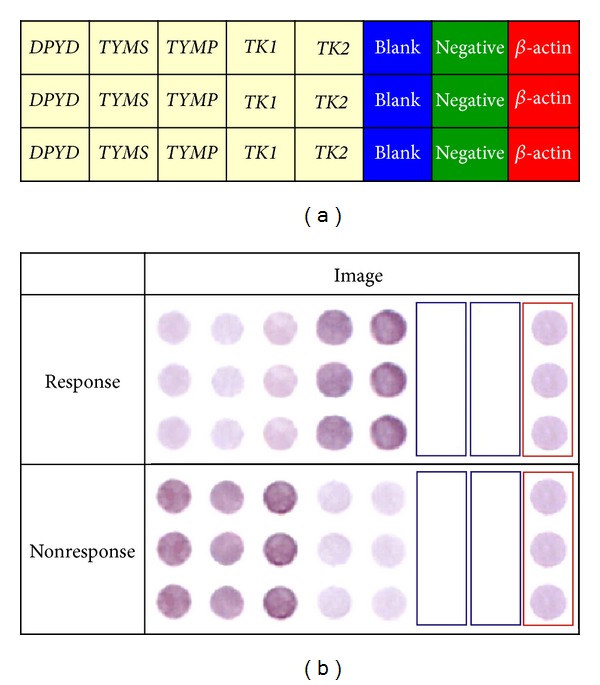
Schematic representations of weighted enzymatic chip array and gene expression patterns of responders and nonresponders. (a) Schematic representation of weighted enzymatic chip array including 5 target genes, one housekeeping gene (*β*-actin), one negative control gene (Negative), and one blank control (Blank). Five target genes (*DPYD*, *TYMS*, *TYMP*, *TK1,* and *TK2*). (b) A triplicate set of 5 genetic biomarkers for locally advanced rectal cancer patients with response and nonresponse is shown on the nylon membrane.

First-strand cDNA targets for hybridization were generated by reverse transcription of the mRNA from the tumor and corresponding normal tissues of LARC patients in the presence of biotin-labeled UTP using the GeneCling Enzymatic Gene Chip Detection Kit. The hybridized arrays were then scanned with an Epson Perfection 1670 flatbed scanner (SEIKO EPSON Corp., Nagano-ken, Japan). Subsequent quantification analysis of intensity of each spot was carried out using AlphaEase FC software (Alpha Innotech Corp., San Leandro, CA, USA). Spots consistently carrying a factor of 2 or more were considered as differentially expressed. A deformable template extracted the gene spots and quantified their expression levels by determining the integrated intensity of each spot after background subtraction. The fold ratio for each gene was calculated as follows: spot intensity ratio = mean intensity of target gene/mean intensity of *β*-actin.  Figure 1 provides the schematic representation of the membrane array with 5 candidate genes, 1 housekeeping gene (*β*-actin), 1 bacterial gene (Mycobacterium tuberculosis; TB), and the blank control (dimethyl sulfoxide; DMSO).

### 2.6. Weighted Enzymatic Chip Array (WEnCA) Analysis

The procedure of the membrane array method for gene detection was performed based on our previous work [32]. Visual OMP3 (Oligonucleotide Modeling Platform, DNA Software, Ann Arbor, MI, USA) was used to design probes for target genes and *β*-actin, and the latter served as an internal control (Table 1). The newly synthesized oligonucleotide fragments were dissolved in distilled water to a concentration of 100 mM and applied to a BioJet Plus 3000 nL dispensing system (BioDot Inc., Irvine, CA, USA), which blotted the target oligonucleotide; the *β*-actin control was used sequentially (0.05 *μ*L per spot and 1.5 mm between spots) on a SuPerCharge nylon membrane (Schleicher and Schuell, Dassel, Germany) in triplicate. DMSO was also dispensed onto the membrane as a blank control. After rapid drying and cross-linking procedures, the preparation of the membrane array was accomplished. The expression levels of each gene spot measured by the WEnCA method were quantified and then normalized based on reference gene (*β*-actin) density. When the normalized spot density was 2 or greater, it was defined as an overexpressed gene spot.

### 2.7. Receiver-Operating Characteristic Curves

Receiver-operating characteristic (ROC) curves were constructed by plotting all possible sensitivity/specificity pairs for the WEnCA analysis, resulting from continuously varying the cutoff values over the entire range of results obtained. According to the analysis of ROC curves, the optimal cutoff point for the number of CCRT response-related genes was obtained. At this cutoff point, the sensitivity and specificity of a panel of multiple genetic biomarkers would also achieve optimal levels. Based on the calculated cutoff values, genetic biomarker panel results were interpreted as either positive or negative chip results.

### 2.8. Statistical Analysis

All statistical analyses were performed using the Statistical Package for the Social Sciences software, Version 14.0 (SPSS Inc., Chicago, IL, USA). ROC curve analyses were performed to analyze the membrane array data of the expression levels of the 9 candidate genes in the tissues of the subjects. The area under the ROC curve (AUC) and the corresponding 95% confidence intervals (CI) were calculated for each gene. The cutoff value at the highest accuracy (with minimal false-negative and false-positive results) was determined. On the basis of the calculated cutoff values, test results were classified as either positive or negative. The sensitivity and specificity of these dichotomous test results and the corresponding 95% CI were determined. A two-sided Pearson Chi-square test and the Fisher exact test were used to analyze the potential correlation between the CCRT response and the clinicopathological features of the study subjects. The multivariate analysis of independent prognostic factors for CCRT response was determined using logistic regression analysis. DFS rates were calculated using the Kaplan-Meier method, and the differences in survival rates were analyzed using the log-rank test. A probability of less than 0.05 was considered statistically significant.

## 3. Results

### 3.1. Constructing a Panel of Multiple Genetic Biomarkers

**Figure 2:**
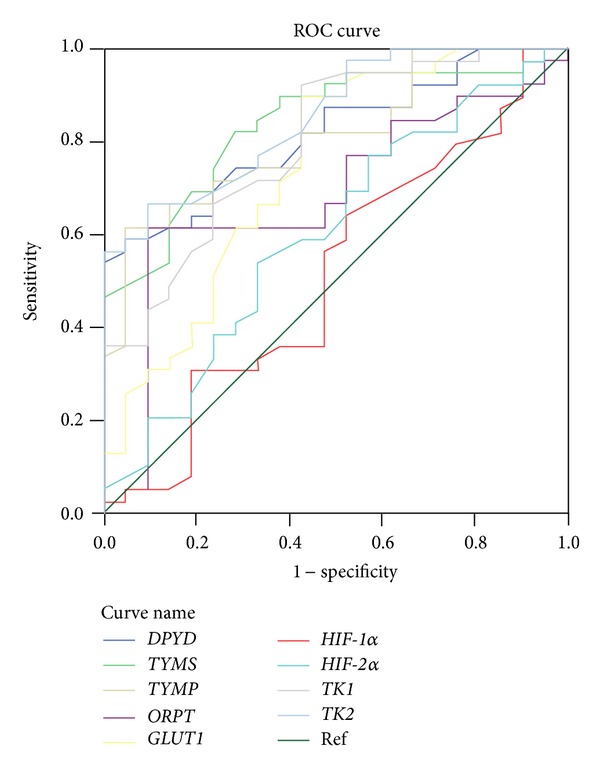
Receiver-operating characteristic (ROC) curves analysis of nine genes and tumor regression grade in 60 rectal cancerous tissues.

The study used the predictive biomarker panel, including 6 genes related to fluoropyrimidine-based chemotherapy (*DPYD*, *TYMS*, *TYMP*, *TK1*, *TK2*, and *ORPT*) and 3 genes related to the radiotherapy response (*GLUT1*, *HIF1*, and *HIF2*). According to ROC curve analysis between all 9 genes and TRG (Figure 2 and Table 2), the cutoff values of the 9 genes (*DPYD*, *TYMS*, *TYMP*, *ORPT*, *GLUT1*, *HIF-1*α**, *HIF-2*α**, *TK1*, and *TK2*) were 2.885, 2.155, 3.215, 2.330, 2.940, 2.455, 3.910, 1.955, and 3.065, respectively, while the sensitivity of each individual gene was above 70% in predicting the CCRT response. We further selected 5 genes with corresponding specificities above 70%, including *DPYD*, *TYMS*, *TYMP*, *TK1*, and *TK2*, to construct a panel of genetic biomarkers for predicting CCRT response (Figure 1). The definition of positive interpretation for each gene was as follows: *DPYD* gene expression less than 2.885, *TYMS* less than 2.155, *TYMP* less than 3.215, *TK1* gene expression more than 1.955, and *TK2* more than 3.065.

### 3.2. ROC Curve Analysis of the Multiple Genetic Biomarker Panel

**Figure 3:**
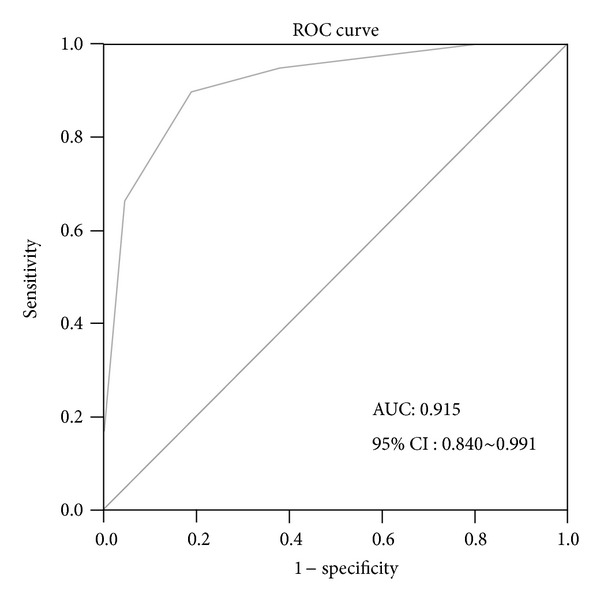
Receiver-operating characteristic (ROC) curve analysis of weighted enzymatic chip array in 60 rectal cancer tissues. In ROC curve analysis of 5 target genes, at a cutoff value of 3 positive genes, a sensitivity of 89.7% and specificity of 81% were obtained (area under ROC curve (AUC): 0.915; and the corresponding 95% confidence intervals (CI): 0.840–0.991) were considered positive results.

From the results of ROC curve analysis of the multiple genetic biomarker panel and TRG, we found that the best cutoff value was 3 genes. In other words, a multiple genetic biomarker panel, on which no less than 3 genes were interpreted as positive, was considered to be positive. The multiple genetic biomarker panel, can predict CCRT response with a sensitivity of 89.7% and a specificity of 81% (AUC: 0.915; 95% CI: 0.840–0.991; Figure 3).

### 3.3. Correlation between Clinicopathological Features/Chip and CCRT Response

Sixty LARC patients (34 men and 26 women; mean age: 63.08 ± 12.71 years) were analyzed, and these patients' characteristics and clinicopathological findings are listed in Table 3. After preoperative CCRT, 39 patients (65%) achieved a pathological tumor response (TRG 2–4). The T classification was downstaged in 29 patients (48.3%), and the N classification was downstaged in 34 patients (56.7%). Univariate analysis indicated that negative perineural invasion (*P* = 0.022) was significantly associated with higher tumor response (Table 3) but that perineural invasion was insignificant in multivariate analysis (*P* = 0.056) (Table 4). Univariate or multivariate analysis indicated that the pre-CCRT CEA level (>2.5 ng/mL versus ≤2.5 ng/mL; >5 ng/mL versus ≤5 ng/mL) was not significantly associated with the CCRT tumor response rate (Tables 3 and 4). Other variables, including age, gender, tumor size, stage, clinical T classification, clinical N classification, differentiation, distance to anus, vascular invasion, and type of chemotherapy, were also not significantly associated with the rate of tumor response. For the correlation between multiple genetic biomarker panel (chip) results and CCRT response, negative chip results were more significantly correlated than positive chip results to poor CCRT responses (TRG 0-1; *P* < 0.001 in univariate analysis and *P* = 0.014 in multivariate analysis; Tables 3 and 4).

### 3.4. Correlation between Multiple Genetic Biomarker Panel (Chip) Results and Disease-Free Survival

**Figure 4:**
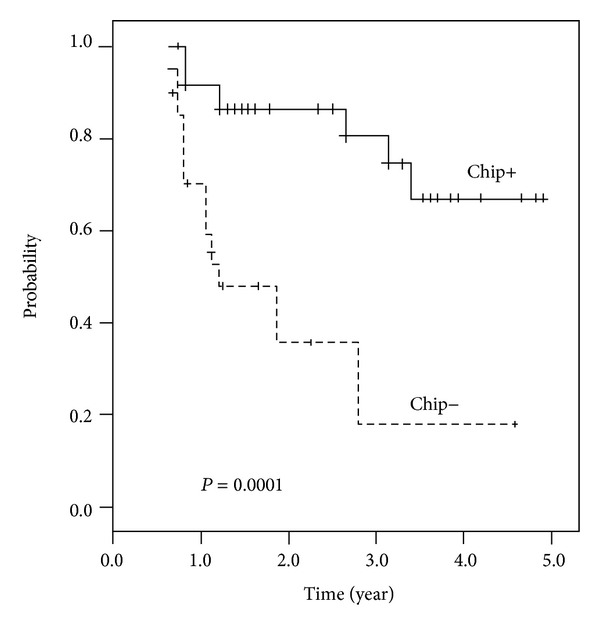
Disease-free survival analysis of 60 locally advanced rectal cancer patients according to results of a multiple genetic biomarkers panel. The median survival rate was 47.01 months in 60 locally advanced rectal cancer patients with positive results, while the median survival rate was only 22.16 months in patients with negative results (*P* = 0.0001).

The median DFS was 47.01 months in patients with positive chip results; on the other hand, the median DFS was 22.16 months in patients with negative chip results (*P* < 0.001; Figure 4).

## 4. Discussion

Preoperative infusional 5-FU and concurrent RT, followed by total mesorectal excision, are the current standard of care for LARC [2]. As compared to postoperative 5-FU based CCRT, this preoperative strategy is associated with significantly lower toxicity and better compliance [2]. A large randomized phase II clinical trial has also provided convincing evidence that preoperative CCRT of rectal cancer reduces local recurrence (6% after 5 years) as compared to postoperative (13% after 5 years) multimodality treatment [2]. However, not all tumors respond uniformly, and despite promising results, *a priori* resistance to CCRT poses a thorny problem, since patients with nonresponsive tumors might either be spared the possible side effects of cytotoxic treatment and radiation or be subjected to alternative treatment modalities [33, 34]. Despite the well-known benefits of neoadjuvant CCRT for LARC, approximately 40% of patients have a poor response to this treatment, due to being exposed to unnecessary toxicities and delays in surgical intervention [7].

The factors predicting response to preoperative CCRT in rectal cancer have not been well characterized. Knowledge of such factors may be useful to clinicians and patients for predicting outcomes and thereby making treatment decisions. A better understanding of predictive factors may eventually lead to the development of such risk-adapted treatment strategies as more aggressive preoperative regimens in patients less likely to respond to standard therapy. Better knowledge of these predictive factors may also help in the design of clinical trials for newer preoperative regimens.

A retrospective study of 141 patients has demonstrated that pretreatment CEA levels greater than 5 ng/mL are associated with poor response to preoperative CCRT [35]. Das et al. reported that pretreatment serum CEA levels greater than 2.5 ng/mL (*P* = 0.015) were associated significantly with lower pathologic complete response rates [36]. Moreno García et al. have reported that pretreatment CEA levels below or equal to 2.5 ng/mL correlate with higher complete pathologic response (21 versus 9%; *P* = 0.05) [37]. However, the study results indicated that pretreatment CEA levels cannot predict CCRT response with either univariate or multivariate analysis, whether the cutoff value of CEA levels was 2.5 ng/mL or 5 ng/mL.

This study has attempted to move beyond single gene expression to a more comprehensive investigation of multiple gene expression levels in predicting tumor response following fluoropyrimidine-based CCRT. The initial investigation involved the expression levels of 9 functional genes; subsequently, a panel of multiple genetic biomarkers was constructed, including the following 5 genes: *DPYD*, *TYMS*, *TYMP*, *TK1*, and *TK2*. In the present study, the RT response-related genes could not well predict response in LARC following fluoropyrimidine-based CCRT. We hypothesis it result from the RT dose (45 Gy) in preoperative CCRT was lower than the definite RT dose (more than 60 Gy). Therefore, the response predictive value of these RT response-related genes (*GLUT1*, *HIF1*, and *HIF2*) could not be highlighted in the adjuvant role.

TRG was reported to have prognostic value in LARC patients after preoperative CCRT and has also been previously reported as an independent prognostic factor for either local recurrence or DFS [7, 38, 39]. Following preoperative CCRT, TRG may reflect the characteristics of proliferation and resistance to hypoxia of residual cancer cells [40]. In our study, 65% of the 60 LARC patients achieved TRG grades 2~4. On comparing Taiwanese patients with other races with regard to tumor response, Berho et al. reported that, of 86 LARC patients receiving preoperative infusional 5-FU and RT, 73.3% of the Caucasians among them achieved a TRG grade between 2 and 4 [41]. These differences in tumor response may explain the variety of CCRT-related responses that occur worldwide. By analyzing multiple gene expression results and TRG, the prediction efficacy of this multiple genetic biomarker panel was demonstrated.

Fluoropyrimidines are antimetabolite drugs widely used in the treatment of solid tumors including rectal cancer [42]. The principal mechanism of action of fluoropyrimidines has been considered to be the inhibition of *TYMS*, but recent evidence has also shown alternative pharmacodynamic pathways acting through the incorporation of fluoropyrimidine's metabolites into the DNA and RNA of tumors [43, 44]. The fluoropyrimidines are broken down into three metabolites that have pharmacodynamic effects, including fluorodeoxyuridine monophosphate (FdUMP), fluoro-deoxyuridine triphosphate (FdUTP), and fluorouridine triphosphate (FUTP). The main mechanism of 5-FU activation is the conversion to FdUMP, which inhibits the enzyme *TYMS*, an important part of the folate-homocysteine cycle and purine and pyrimidine synthesis [43]. The conversion of 5-FU to FdUMP can occur via *TYMP* to fluorodeoxyuridine and then by the action of thymidine kinase to FdUMP or indirectly by fluorouridine monophosphate (FUMP) or fluorouridine (FUR) to fluorouridine diphosphate (FUDP) and then ribonucleotide reductase action to fluorodeoxyuridine diphosphate and FdUMP [43]. The incorporation of dUTP or FdUTP into DNA is the cause of DNA damage of tumor cells of fluoropyrimidines [44]. The rate-limiting step of 5-FU catabolism is *DPYD* conversion of 5-FU to dihydrofluorouracil [45]. To modulate the activity of fluoropyrimidines, inhibitors of *DPYD*, such as uracil and eniluracil, can be coadministered. This slows the degradation of 5-FU and improves the response rate [43]. Meanwhile, metabolites of fluoropyrimidine are crucial for LARC therapy.

The main enzymes implicated in fluoropyrimidine metabolism have been widely studied for response prediction. It has been established that higher *TYMP* (as well as lower *DPYD*) expression in tumors resulted in higher intratumoral concentrations of 5-FU, as well as a more potent antitumor effect of capecitabine [42–44]. In line with this idea, positive immunostaining for *TYMP* has predicted a significantly higher response rate to a capecitabine regimen in advanced stages of CRC [45]. Likewise, Boskos et al. have found that patients with a higher *TYMP*/*DPYD* ratio by ELISA were more likely to respond to neoadjuvant capecitabine/RT [21]. Thymidylate synthase (*TYMS*) is considered the indirect target of 5-FU. High *TYMS* expression in pretreatment biopsies, measured either by IHC [46] or by mRNA [12], has been linked to a lack of response to neoadjuvant 5-FU/RT. The number of tandem repeats in the *TYMS* promoter region affects the translation efficiency of the protein, leading to increased expression [42]. Patients with triple repeats of this sequence (*TYMS* 3/3) had poorer tumor responses than those with shorter sequences (2/2 or 2/3) [43], suggesting that germline analysis for genetic variants may assist in predicting response. However, these studies are hypothesis-generating and, to date, there are neither studies confirming them nor contradictory findings [14]. In the current study, simultaneous positive interpretations of 3 genes (out of the 5) can predict fluoropyrimidine-based CCRT response with a sensitivity of 89.7% and specificity of 81%. The identification of predictive indicators of CCRT would be extremely useful in selecting feasible patients for fluoropyrimidine-based preoperative CCRT, thereby avoiding unnecessary preoperative treatment. In the present study, the median DFS was 47.01 months in LARC patients with positive chip results; on the other hand, the rate was only 22.16 months in patients with negative results. There were prominent associations between chip results and DFS, which could be used as a pre-CCRT predictor for clinical outcomes of LARC. The study data suggest that positive chip results might be predictors not only of tumor response but also of DFS. This finding could be useful in the future to identify individual risk and to develop more aggressive or alternative therapeutic strategies.

In conclusion, the present study indicates that a panel of multiple genetic biomarkers, consisting of the *DPYD*, *TYMS*, *TYMP*, *TK1*, and *TK2* genes, could be a potential aid in clinical predictions to obtain better CCRT response prediction models. It suggests that such a panel could be used to distinguish between LARC patients responding to CCRT and those who do not. Moreover, further studies in larger sample sizes and even multiple centers are mandatory to verify these results.

## Figures and Tables

**Table 1:** Oligonucleotide sequences of target genes and the *β-actin* gene.

Gene name	Oligonucleotide sequence
*DPYD *	CAGTCAGAGCCCGTATGTGCACAGCAAAAGAGTGGTAACCAGGATCTATC
*ORPT *	GCTGCTGAGATTATGCCACGACCTACAATGATGATATCGGAACCTCGTTT
*TYMS *	GGATCCCTTGATAAACCACAGCAACTCCTCCAAAACACCCTTCCAGAACA
*TYMP *	CATCTGCTCTGGGCTCTGGATGACATTGAATCCAGGAATAGACTCCAGCT
*TK1 *	AAGGTTGGTGCCACCCATCTTGGTGAAAGATGCTGTTGTTCCTGTGGAAA
*TK2 *	CACTGAACACCGGGCTCCAGCCAAATGCAGCATAATTTTGTGGAAGTCTA
*GLUT1 *	CAACCCCACTTACTTCTGTCTCACTCCCATCCAAACCTCCTACCCTCAAT
*HIF-1 α*	GTTCTATGACTCCTTTTCCTGCTCTGTTTGGTGAGGCTGTCCGACTTTGA
*HIF-2 α*	TCAGTGCTTCCTACCTACATGTCACTGACCGACCCAGAGACCTCAGCCAG
*β-actin *	TCATGAAGTGTGACGTGGACATCCGCAAAGACCTGTACGCCAACACAGTGCTGTC

**Table 2:** The area under the ROC curve (AUC) and the corresponding 95% confidence intervals (CI) of 9 genes.

Gene	AUC	95% CI
*DPYD *	0.815	0.711–0.919
*TYMS *	0.836	0.734–0.939
*TYMP *	0.81	0.701–0.918
*ORPT *	0.683	0.54–0.827
*GLUT1 *	0.743	0.605–0.881
*HIF-1 α*	0.514	0.355–0.673
*HIF-2 α*	0.601	0.449–0.754
*TK1 *	0.798	0.682–0.914
*TK2 *	0.852	0.759–0.946

**Table 3:** Correlations between clinicopathological features and response status in 60 locally advanced rectal cancer patients.

Characteristics	Total cases	Nonresponse	Response	*P* value
	*n* (%)	*n* (%)	*n* (%)	
Gender				
Female	26 (43.3%)	7 (33.3%)	19 (48.7%)	0.251
Male	34 (56.7%)	14 (66.7%)	20 (51.3%)	
Age (years)				
<60	22 (36.7%)	8 (38.1%)	14 (35.9%)	0.886
≥60	38 (63.3%)	13 (61.9%)	25 (64.1%)	
Tumor size				
<5 cm	46 (76.7%)	14 (66.7%)	32 (82.1%)	0.179
≥5 cm	14 (23.3%)	7 (33.3%)	7 (17.9%)	
Stage (UICC)^a^				
II	12 (20.0%)	5 (23.8%)	7 (17.9%)	0.737
III	48 (80.0%)	16 (76.2%)	32 (82.1%)	
Clinical T-stage				
T3	54 (90.0%)	19 (90.5%)	35 (89.7%)	1.000
T4	6 (10.0%)	2 (9.5%)	4 (10.3%)	
Clinical N-stage				
N0	12 (20.0%)	5 (23.8%)	7 (17.9%)	0.846
N1	23 (38.3%)	8 (38.1%)	15 (38.5%)	
N2	25 (41.7%)	8 (38.1%)	17 (43.6%)	
Chemotherapy				
Capecitabine	36 (60.0%)	10 (47.6%)	26 (66.7%)	0.151
5-FU	24 (40.0%)	11 (52.4%)	13 (33.3%)	
Time intervals of CCRT to operation				
<6 weeks	20 (33.3%)	8 (38.1%)	12 (30.8%)	0.566
(5.35 ± 0.49 weeks)				
≥6 weeks	40 (66.7%)	13 (61.9%)	27 (69.2%)	
(7.15 ± 1.20 weeks)				
Differentiation^b^				
WD	2 (3.3%)	0 (0.0%)	2 (5.1%)	0.653
MD	47 (78.3%)	17 (81.0%)	30 (76.9%)	
PD	4 (6.7%)	2 (9.5%)	2 (5.1%)	
Unclassified	7 (11.7%)	2 (9.5%)	5 (12.8%)	
Distance to anus				
<5 cm	38 (63.3%)	11 (52.4%)	27 (69.2%)	0.196
≥ 5 cm	22 (36.7%)	10 (47.6%)	12 (30.8%)	
Vascular invasion				
Yes	52 (86.7%)	18 (85.7%)	34 (87.2%)	1.000
No	8 (13.3%)	3 (14.3%)	5 (12.8%)	
Perineural invasion				
Yes	40 (66.7%)	10 (47.6%)	30 (76.9%)	0.022
No	20 (33.3%)	11 (52.4%)	9 (23.1%)	
Pre-CCRT CEA				
>2.5 ng/mL	44 (73.3%)	15 (71.4%)	29 (74.4%)	0.807
≤2.5 ng/ml	16 (26.7%)	6 (28.6%)	10 (25.6%)	
Pre-CCRT CEA^c^				
>5 ng/mL	27 (45.0%)	10 (47.6%)	17 (43.6%)	0.765
≤5 ng/mL	33 (55.0%)	11 (52.4%)	22 (56.4%)	
Chip^d^ result				
Negative	21 (35.0%)	17 (81%)	4 (10.3%)	<0.001
Positive	39 (65.0%)	4 (19%)	35 (89.7%)	

^a^UICC: The American Joint Commission on Cancer/International Union Against Cancer (AJCC/UICC, 2002).

^b^WD: well differentiated, MD: moderately differentiated, PD: poorly differentiated.

^c^CEA: carcinoembryonic antigen.

^d^Chip: panel of multiple genetic biomarkers.

**Table 4:** Univariate and multivariate regression analysis of prognostic indicators and nonresponse status for 60 locally advanced rectal cancer patients.

Parameters	Number	Univariate analysis	*P* value	Multivariate analysis	*P* value
		Hazard ratio (95% CI)		Hazard ratio (95% CI)	
Sex (female/male)	26/34	0.654 (0.309–1.384)	0.251	0.088 (0.005–1.613)	0.102
Age (≥60/<60)	38/22	0.941 (0.464–1.908)	1.000	0.093 (0.003–2.979)	0.179
Tumor size (≥5 cm/<5 cm)	14/46	1.643 (0.831–3.250)	0.179	4.658 (0.145–50.100)	0.385
Stage (UICC)^a^ (II/III)	12/48	1.250 (0.573–2.727)	0.737	7.244 (0.114–458.695)	0.349
Clinical T-stage (T4/T3)	6/54	0.947 (0.289–3.108)	1.000	0.752 (0.023–24.539)	0.873
Clinical N-stage (N2/N1 + N0)	25/35	0.862 (0.421–1.762)	0.681	0.396 (0.011–14.706)	0.616
Chemotherapy (capecitabine/5-FU)	36/24	0.606 (0.306–1.200)	0.151	0.061 (0.002–1.745)	0.102
Differentiation^b^ (PD + MD/WD)	51/2	0.627 (0.508–0.775)	0.531	0.000 (0.000–0.000)	0.999
Distance to anus (<5 cm/≥5 cm)	38/22	0.637 (0.324–1.253)	0.196	0.107 (0.002–5.908)	0.275
Vascular invasion (Yes/No)	52/8	0.923 (0.350–2.434)	1.000	2.022 (0.084–48.564)	0.664
Perineural invasion (Yes/No)	40/20	0.455 (0.233–0.886)	0.022	0.043 (0.002–1.0780)	0.056
Pre-CCRT CEA^c^ (ng/mL) (>2.5/≤2.5)	44/16	0.909 (0.428–1.933)	0.807	0.314 (0.004–23.578)	0.599
Pre-CCRT CEA^c^ (ng/mL) (>5/≤5)	27/33	1.111 (0.558–2.213)	0.765	0.407 (0.018–8.967)	0.569
Chip^d^ result (negative/positive)	21/39	7.893 (3.049–20.434)	<0.001	22.704 (3.055–235.448)	0.014

^a^UICC: The American Joint Commission on Cancer/International Union Against Cancer (AJCC/UICC, 2002).

^b^WD: well differentiated, MD: moderately differentiated, PD: poorly differentiated.

^c^CEA: carcinoembryonic antigen.

^d^Chip: panel of multiple genetic biomarkers.
